# The Role of the Molecular Clock in Promoting Skeletal Muscle Growth and Protecting against Sarcopenia

**DOI:** 10.3390/ijms20174318

**Published:** 2019-09-03

**Authors:** Jacopo A. Vitale, Matteo Bonato, Antonio La Torre, Giuseppe Banfi

**Affiliations:** 1 IRCCS Istituto Ortopedico Galeazzi, LaMSS—Laboratory of Movement and Sport Science, Via Riccardo Galeazzi 4, 20161 Milano, Italy; 2 Department of Biomedical Sciences for Health, Università degli Studi di Milano, Via Giuseppe Colombo 71, 20133 Milano, Italy; 3 Vita-Salute San Raffaele University, via Olgettina 58, 20132 Milano, Italy

**Keywords:** Sarcopenia, muscle, strength, function, ageing, orthopaedics

## Abstract

The circadian clock has a critical role in many physiological functions of skeletal muscle and is essential to fully understand the precise underlying mechanisms involved in these complex interactions. The importance of circadian expression for structure, function and metabolism of skeletal muscle is clear when observing the muscle phenotype in models of molecular clock disruption. Presently, the maintenance of circadian rhythms is emerging as an important new factor in human health, with disruptions linked to ageing, as well as to the development of many chronic diseases, including sarcopenia. Therefore, the aim of this review is to present the latest findings demonstrating how circadian rhythms in skeletal muscle are important for maintenance of the cellular physiology, metabolism and function of skeletal muscle. Moreover, we will present the current knowledge about the tissue-specific functions of the molecular clock in skeletal muscle.

## 1. Introduction

Recently it has been observed that skeletal muscle exhibits a variety of behavioural, physiological and biochemical variations that occur over the course of the day/night cycle [1]. These rhythms, termed “circadian”, comprise a series of interconnected transcriptional feedback loops that optimize the timing of cellular events in anticipation of environmental conditions [2]. The internal master circadian clock resides within the suprachiasmatic nuclei (SCN) of the anterior hypothalamus [3] and autonomous circadian clocks are present in all cells of the body, including skeletal muscle cells.

Skeletal muscle comprises approximately 40% of the total body mass; it is therefore the most abundant tissue in the body and one of the main components of the locomotor apparatus [4]. It is composed of more than 600 muscles, with different fibre-type compositions, metabolic capacities, and mechanical functions that consequently produce forces and enable locomotion [5]. In addition to this, skeletal muscle is the main reserve for amino acids in the absence of nutrient intake, allowing the maintenance of protein synthesis in other tissues of the body. Muscle functions are thus critical for body health and their deterioration can lead to the development of disease.

Aging is responsible for many changes in body composition, including a reduction of muscle mass [6]. According to Deschenes et al. [7] from the age of 25 there is a progressive reduction in the size and number of muscle fibres resulting in a total decrease of about 40% in muscle mass. Furthermore, it was demonstrated that the turning point between muscle gain and muscle loss occurs around 27 years [8]. In recent years, a large percentage of individuals have shown an accentuated muscle loss, diminished strength and impaired muscle metabolism that can lead to a significant increased frequency of subsequent adverse health outcomes (e.g., physical frailty, falls, fractures, disability etc.) [9,10]. This progressive reduction of muscle mass, muscle strength and function that occurs with aging leads to a pathological functional impairment that has been defined with the term “sarcopenia” (Greek, Sarx for “flesh” and Penia for “loss”) [11].

Maintenance of circadian rhythms is emerging as an important new factor in human health with disruptions linked to ageing as well as to the development of many chronic diseases, including sarcopenia. Therefore, the aim of this review is to present the latest findings demonstrating how circadian rhythms in skeletal muscle are important for maintenance of the cellular physiology, metabolism and function of skeletal muscle. Moreover, we will present the current knowledge about the tissue-specific functions of the molecular clock in skeletal muscle.

## 2. Sarcopenia: The Loss of Muscle Mass, Strength and Function

Generally, sarcopenia is characterized by a progressive reduction in skeletal muscle mass and quality [11]. In particular, it has been observed that there is replacement of muscle fibres with fat over the time, with a progressive increase in fibrosis, changes in muscle metabolism, oxidative stress and degeneration in the neuromuscular junction that ultimately leads to frailty [12]. From a histological point of view the sarcopenic status predominantly affects type II muscle fibres that can be reduced up to 50, whereas type I fibres are less affected [13]. Since these reductions are only moderate when compared to the overall decrease in muscle mass it has therefore been stated that sarcopenia represents both a reduction in muscle fibre quantity and size [11]. To demonstrate this, Lexell et al. [14], compared muscle cross-sections and observed that elderly subjects in the ninth decade had 50% less type I and type II fibres that younger people. Moreover, evidence from anatomical and electrophysiological studies demonstrates a marked loss of anterior horn cells and ventral root fibres that occurs with ageing [15,16]. From a molecular point of view, changes in myofibers is caused also by the increase of insulin-like growth factor 1. In fact, it activates PI3K/Akt signalling and increases the expression of Atrogin-1 and the muscle ring finger protein 1 (muscle-specific E3 ligases that play a key role in regulating ubiquitin-driven protein degradation in skeletal muscle tissues, via the suppression of forkhead box transcription factors). In addition, this activation of PI3K/Akt signalling has been shown to result in an increase in protein synthesis, by activating the mechanistic target of rapamycin (mTOR) complex and inhibiting glycogen synthase kinase (GSK)-β [17]. In addition, myostatin, activin A, and transforming growth factor (TGF)-β, bind activin type 2 receptor B (ActR2B), and this contributes to inducing sarcopenia, by activating Smad2/3, which reduces the phosphorylation of Akt [18]. Furthermore, the apoptosis of myofibers is considered as another mechanism of sarcopenia. Apoptotic loss of skeletal muscle mass can lead to lipid droplet accumulation in skeletal muscle tissues, which is associated with poor skeletal muscle function. The sarcopenic process can be also accelerated by numerous factors, including nutritional deficiencies, lack of physical exercise, hormonal changes, metabolic disturbance, comorbidities, inflammation, drug adverse effects, genetic predisposition and the effect of early environments [19].

For these reasons, sarcopenia is associated with functional disabilities, quality-of-life impairments, falls, osteoporosis, dyslipidaemia, an increased cardiovascular risk, metabolic syndrome and immunosuppression [10], consequently leading to an increase in morbidity. In fact, it has been observed that both muscle mass and muscle function are independently associated with a 3.7-fold increase in mortality [20,21,22], a two-fold increase in risk of falling [23], as well as with a greater risk of dependency [24]. Moreover, sarcopenia is associated with a 50% increase in the risk of admission, a 20-day increase in hospital stay length, and a 34% to 58% increase in hospital care cost [25,26].

A large variability in the prevalence of sarcopenia is observed in the general population. von Haehling et al. [27] stated that the 5%–13% of 60 to 70-year-old people and 11%–50% of those older than 80 years are in a sarcopenic status. More specifically, Patel et al. [28] observed a prevalence of sarcopenia in older adults of 4.6% in men and 7.9% in women; Brown et al. [29] observed 36.5%; Kim et al. [30] observed from 2.5% to 28.0% in men and 2.3% to 11.7% in women; and Wu et al. [31] observed from 3.9% to 7.3%.

Early recognition of sarcopenia is therefore essential. Screening patients for impairment in their physical function should be routine with subsequent, more specific testing for sarcopenia. Several objective methods for evaluating sarcopenia are currently used including calf circumference, bio-impedance analysis (BIA), dual-energy X-ray absorptiometry (DEXA), computerized tomography, magnetic resonance imaging, hand-grip strength, and walking speed [32]. However, none of these measures are considered the gold standard for evaluating sarcopenia [33]. In 1998, Baumgartner and colleagues [34] proposed using DEXA to assess lean body mass, comparing it to gender specific healthy young adults with a cut-off point of two standard deviations below the mean of lean mass. However, this method is not able to distinguish water retention or fat infiltration within muscle or the muscle mass in relation to total body mass. Since sarcopenia encompass both quantitative (i.e., muscle mass) and qualitative (i.e., strength and/or function) decline in skeletal muscle, several definitions of sarcopenia have been proposed [21,34,35,36,37,38]. For that reason, the European Working Group on Sarcopenia in Older People (EWGSOP) proposed in 2010 the following criteria for diagnosing sarcopenia [11]: (i) Low muscle mass: Assessed by skeletal mass index with threshold levels of ≤8.90 kg/m^2^ for men and ≤6.63 kg/m^2^ for women; (ii) Low muscle strength: Assessed with handgrip strength with threshold levels of <30 kg for men and 20 kg for women; and (iii) Low physical performance: Assessed by gait speed of ≤0.8 m/s during a six minute walking test. It is important to note that sarcopenia is now recognized as an independent condition by an ICD-10-CM code [39].

## 3. Human Biological Rhythms

Chronobiology is the science that objectively quantifies the biological mechanisms of time structures. The expression of biological rhythms seems to be crucial for body homeostasis, as individuals perform optimally when all biological rhythms are synchronized [40]. Human rhythms are present at all levels of biologic integration and they are typically classified in three categories: i) Circadian rhythms: A period between 20 h and 28 h [40,41]; ii) Ultradian rhythms: A period <20 h [40,41]; and iii) Infradian rhythms: A period >28 h, including circaseptan and circannual rhythms [41,42]. Each biological rhythm is characterized by: i) The acrophase, which indicates the time interval within which the highest values are observed; ii) The amplitude, a measure of one half of the extent of rhythmic variation in a cycle; and iii) The MESOR (midline estimating statistic of rhythm), the mean of a rhythmic function [42,43]. Therefore, human circadian rhythms are affected by the interaction of several exogenous, endogenous, and lifestyle mechanisms that work at the same time [44]. The internal master circadian clock is located within the suprachiasmatic nuclei (SCN) of the anterior hypothalamus [3], with autonomous circadian clocks that are also present in nearly all tissues [45]. In particular, the SCN is also synchronized with the external environment by exogenous factors called “synchronizers” (or “zeitgebers”, i.e., “time-givers” in German). The primary synchronizer for the human body clock is the light–dark cycle that provides information to the SCN, through the retinohypothalamic tract, leading to a synchronization of the peripheral clocks by neuro-hormonal signalling [46]. The secondary synchronizers are physical activity, sleep and meal timing, and social routine [47,48].

The peripheral clocks regulate many functions of tissues and they contribute to their homeostasis [46,49]. At the molecular level, a complex transcriptional–translational network of the circadian clock circuit, which is able to generate circadian rhythmicity, has been described in detail, although new modulators of the circadian clock loop continue to emerge [50,51]. The molecular clock is based on the activity of two positive elements, clock (circadian locomotor output cycles kaput) and *Bmal*1 (brain and muscle Arnt-like protein 1). Their dimerization activates the rhythmic transcription of *Per* and *Cry* genes. *Per* and *Cry* proteins dimerize and migrate to the nucleus where they inhibit the Clock–*Bmal*1 dimerization, resulting in the inhibition of *Per* and *Cry* transcription in a process that takes approximately 24 h for a complete feedback cycle [51]. This molecular clock regulates the expression of several clock-controlled genes which are responsible for the rhythmicity of many physiological processes [1] and this highlights that the components of the molecular clock work together to coordinate the circadian rhythmicity of the peripheral tissue-specific genes, including those in the skeletal [52,53].

## 4. The Molecular Clock in Skeletal Muscle

Despite the intrinsic mechanism being the same across cell types, the molecular clock output is highly tissue-specific [54] and it has been observed that it acts in skeletal muscle as well as in bone [51,55]. Skeletal muscle, which represents the most abundant tissue in mammals (≃40% of body mass), possesses a self-sustaining endogenous molecular clock [56].

### Skeletal Muscle Growth and Maintenance

Previous studies performed on mice with clock genes mutations have demonstrated a clear link between the maintenance of skeletal muscle development and growth and the molecular clock circuit as a whole [57]. To deeply understand the role of the molecular clock in peripheral tissues, the use of genetic mouse models targeting the loss of *Bmal*1 have been adopted [58]. It has been shown that the loss of *Bmal*1 leads to severe aging-associated sarcopenia: The genetic loss of *Bmal*1 at 9 months of age, resulted in the decrease of about half of the body weight, indicating a premature aging phenotype [59]. However, recent findings highlight that the intrinsic muscle clock is not essential for muscle growth and its inactivation does not lead to reduced life span and premature aging [60]. It has been reported that muscle phenotype, in different models of *Bmal*1 knockout (KO), is variably affected; the muscle loss and early ageing process observed in the global conventional KO are not present in muscle-specific *Bmal*1 KO [61] or in global KO induced in adults [60], and thus must reflect the loss of *Bmal*1 function during development and in non-muscle tissues [62]. Clock mutant mice showed a clear skeletal muscle weakness, structural muscle pathology and altered mitochondrial volume and myofilament architecture [56]. About 20% of genes show 24 h oscillations in skeletal muscle and about the 30% of those circadian transcripts decrease or lose their rhythmicity in clock-mutant animals [63] and this supports the hypothesis that the molecular clock has a key role in maintaining the correct temporal regulation of clock-controlled genes (CCGs) in muscles. During the first stages of muscle growth and myoblast differentiation, the *MyoD1* (myogenic differentiation 1) is activated and it has emerged as a CCG based on its distinct circadian expression trend in adult muscle [64]. Furthermore, the ablation of clock or *Bmal*1, the key elements of the molecular clock, blunts both MyoD1 circadian expression and its target genes. This consequently leads to a disruption of muscle contractile function and alteration in the organization of the sarcomeric myofibers [63]. Similar results were found in *MyoD1* null mice and, consequently, *Myod1* was reported to be a target of *Bmal*1, and *Myod1* was reported to lose its circadian oscillation in *Bmal*1 KO mice [64]. However, these data are in contrast with a subsequent study that showed *Myod1* gene expression is increased rather than decreased in in muscle-specific *Bmal*1 KO mice and maintains its circadian oscillation [62]. A similar effect, with an even greater upregulation of *Myod1*, was observed after denervation. Based on these results, it seems unlikely that *MyoD* can mediate the effect of *Bmal*1 function on the skeletal muscle.

Since the maintenance of skeletal muscle mass encompasses both the myonuclear accretion in first stages of growth and the mature myofiber hypertrophy in adulthood [65], the muscle loss in adult *Bmal*1-null mice may result from a combination of both components. The surveying of clock-controlled mRNA expression in the skeletal muscle reveals that differentiation and proliferation comprise about 15% of the total transcripts [63] and, in line with these data, it has been demonstrated that regulation of myogenic progenitor cells by *Bmal*1 is essential for the regeneration of muscle tissue after injury [66]. To confirm this, animals without *Bmal*1 showed a defect in regenerative myogenic process accompanied by lowered repair and an altered satellite cell expansion process when evaluated in muscle injury-elicited regeneration models [66]. Muscle growth and homeostasis needs the contribution of muscle satellite cells and their proliferative capacity is constantly reduced with age [67]; the status of sarcopenia observed in *Bmal*1-null mice could be partially mediated by the decline of the clock function.

Cytosolic *Bmal*1 is indicated as a factor, able to facilitate protein translation, that links the circadian network and the mechanistic target of rapamycin (mTOR) signalling pathway [68], which is a one of the major mechanisms of regulation that supports protein synthesis and induces hypertrophy in skeletal muscle [65,69]. It is noteworthy that the mTOR signalling pathway is reported to be under circadian regulation and the *Bmal*1-mediated mTOR circadian modulation of translation activities is controlled by the circadian oscillatory magnesium levels in cells [66,68]. Furthermore, both the clock repressor, *Rev-erbα*, and its reciprocal transcription activator *Rorα* on the *Rore* responsive element are involved in the regulation of myogenic differentiation [70,71]. The loss of Rev-erbα deficient mice leads to a lower body weight and non-regular myosin heavy chain (MyHC) isoform expression, with a fast-to-slow *MyHC* isoform transformation in skeletal muscle. This suggests the involvement of *Rev-erbα* in muscle mass maintenance and metabolic control [71].

## 5. Muscle Strength and Physical Function

The circadian clock in animals has strong control of locomotor activity cycles. The strict behavioural circadian rhythmicity of animals ensures their survival. Humans also perform optimally when all biological rhythms are correctly synchronized [72]. It has been widely demonstrated that human muscle torque, strength and power are higher in the late afternoon than they are in the morning [73,74] and many other metrics of athletic performance, e.g., reaction time, joint flexibility, and sprint ability, display significant circadian variations and time of day dependence [44,75]. Athletes’ knee muscles, evaluated during a maximal isometric strength test, displayed a typical circadian pattern with an acrophase occurring in the mid-to-late afternoon period (16:00–20:00) [76]. Furthermore, a chronotype effect on the circadian expression of many types of physical performance has been recently observed [77,78,79].

Nonetheless, as mentioned above, the molecular clock can be influenced through external manipulations; indeed, it has been demonstrated that scheduled exercise, for instance, is able to shift behavioural rhythms in mammals, specifically the skeletal muscle molecular clock [80,81]. Different sessions of long-term (or endurance) exercise led to a modification in the expression of the clock gene, PER2:LUC bioluminescence, in three different types of muscle as well as in the lungs. Interestingly, this shift in gene expression was only observed in these two tissues and this result clearly suggests that exercise is a non-SCN-associated entrainment cue for skeletal muscle. In addition, the phase of the three selected skeletal muscles was different before exercise, highlighting the complexity and diversity of the skeletal muscle circadian rhythms [80]. This difference could be partially due to the different muscle composition and function: The soleus is a postural muscle, mostly composed of type I slow-oxidative fibres, while the extensors and flexors of the fingers are intermittently recruited during daily activities and they are principally composed of type II fast oxidative-glycolytic fibres. Despite structural baseline muscle differences, authors observed that scheduled activity shifted the phases in the same magnitude, from about 2 to 3 h [80]. Nonetheless, it is essential to highlight that changes in muscle circadian rhythms induced by exercise may be due to a direct effect on the muscle clock but also to systemic effects caused by exercise, such as hormonal changes or variations in body temperature, which are able to secondarily affect the peripheral oscillators, namely the muscle clock [82,83,84]. Sleep and the sleep–wake cycle are also essential to promoting optimal functioning of the molecular clock. It was found that partial sleep deprivation had negative effects on the power output of muscle performances, even if this effect was dependent on the timing and duration of sleep disruption and on the time of the day of the measurements [85]. These results confirm the hypothesis of an intimate interplay between clock control and physical activity and, in line with this statement, it was also reported that the increase in activity levels entrained core clock genes and CCGs in human skeletal muscle [86]. Furthermore, strength exercise showed shifts in the expression of diurnally-regulated genes in the human muscles, both by down-regulating genes that are highly expressed and also by inducing genes that are normally repressed [86]. On the other hand, loss of muscle activity by unilateral sciatic nerve denervation leads to marked atrophy and reduces the expression of *Bmal*1, *Per1*, *Rorα* and *Rev-erbα* in skeletal muscles [87,88]

Overall, given that strength exercise is able to affect the expression of core clock components and downstream targets in human muscles [66,86], the peak expression of rhythmic transcripts in muscle could be attributed to the orchestration of the endogenous muscle clock control and central clock-induced locomotor activity rhythm. It is interesting to note that, although physical exercise can regulate circadian clock genes (*Per2*, *Cry1*, and *Bmal*1) and circadian output genes in skeletal muscles, the SCN rhythms seem to be not affected [80]. The SCN is probably involved in the long-term effects of clock phase shifting in peripheral tissue and the current evidence suggests that the skeletal muscle clock responds more quickly to physical exercise by transcriptional regulation of specific clock genes. These results support the emerging idea that peripheral clocks can regulate themselves independently of the SCN [48,89] even if the underlying physiological mechanisms need to be elucidated.

### Muscle Metabolic Pathways

Skeletal muscle is a predominant contributor to whole-body metabolism and it seems that the molecular clock may regulate the temporal control of metabolic mechanisms [90]. Alterations to this regulatory mechanism significantly disrupt the metabolic homeostasis, possibly determining the development of insulin resistance and obesity [91,92,93], and previous studies in clock mutants report that about 35% of rhythmic genes in skeletal muscle are involved in metabolism [56,66]. Furthermore, clock-controlled regulation of anabolic and catabolic metabolic pathways has been observed in skeletal muscles and a different temporal activation of genes that regulate substrate storage and utilization was revealed over a daily period [94,95]. It is clear that skeletal muscle *Bmal*1 is necessary for maintenance of the skeletal muscle metabolism, particularly glucose handling pathways and a specific *Bmal*1 deletion in skeletal muscle was created to test the function of *Bmal*1 in skeletal muscle glucose metabolism to confirm this [93,94]. The results showed that the muscle-specific deletions of *Bmal*1 lead to an altered insulin-dependent glucose uptake and to a reduction in glucose oxidation [61]. In addition, GLUT4 glucose transporter levels, responsible for glucose uptake, were significantly lower, whereas the insulin signalling pathway was not affected [61]. Furthermore, it was also shown that the deletion of muscle-specific *Bmal*1 may lead to an increase of oxidative capacity in mice, specifically to an increased energy expenditure, oxygen consumption and resistance to obesity with improved metabolic profiles. This mechanism includes the regulation of *Cacna1s* expression, followed by the activation of the calcium—nuclear factor of activated T cells axis and it was concluded that *Bmal*1 is a critical regulator of the muscular fatty acid level under nutrition overloading [96]. Following studies conducted with a mouse model of *Bmal*1 ablation in muscle, revealed abnormal expression of the genes involved in metabolic substrate oxidation [94]: A clear up-regulation of genes involved in lipid metabolism was observed with a concomitant down-regulation of circadian genes involved in glucose utilization. These findings lead to the observation that muscle fiber type switches to a slow oxidative fiber-type, together with a substrate shift from carbohydrate to lipid utilization [94].

*Rev-erbα*, which is the circadian clock repressor gene, plays also key functions in metabolic regulations in the skeletal muscle [97,98,99]. It has been found that *Rev-erbα* is highly expressed in oxidative fiber types and that it also promotes the oxidative capacity in skeletal muscle inhibition of mitochondria autophagy [100]. In *Rev-erbα*-deficient mice, a clear fast-to-slow MyHC isoform transformation was observed, albeit only in soleus muscle [101]. Furthermore, it was observed that a *Rev-erbα*-deficiency resulted in increased fatty acid utilization and hyperglycemia suggesting a preference toward glucose utilization at the expense of peripheral lipid utilization for *Rev-erbα* [100]. Interestingly, the activation of *Rev-erbα* by synthetic agonists, that display lipid lowering and anti-obesity efficacy, demonstrate oxidation of fatty acid pathways while suppressing lipid synthesis genes in skeletal muscle [102]. Overall, the current results suggest that the molecular clock machinery could play an essential role for skeletal muscle metabolism.

## 6. Conclusions

The circadian clock has a critical role in many physiological functions of skeletal muscle and it is essential to fully understand the specific underlying bio-physiological processes that regulate these complex interactions. The importance of circadian expression for skeletal muscle structure, function and metabolism is clear when observing the muscle phenotype in models of molecular clock disruption. To confirm this, the loss of the *Bmal*1 gene leading to sarcopenia and several pathological muscle conditions, has been observed, including effects such as lowered mitochondria density and altered mitochondrial respiration, fibre-type shifts, impaired sarcomeric structure and restricted function [64]. However, it is important to highlight that the rescue of the *Bmal*1 in the skeletal muscle protected animals from the decrease in muscle mass and function [103] and, therefore, this was able to mitigate the pathological mechanisms of the skeletal muscle. An increased entrainment of skeletal muscle circadian rhythms may also contribute to systemic health. These suggests that the correct circadian expression of skeletal muscle may represent a possible target of intervention in many diseases.

It is also necessary to understand how other kinds of clock disruptions, such as the desynchronization between endogenous circadian clock cycles and the exogenous light–dark cycle (commonly identified in our society as “social jetlag”), could affect muscle growth and maintenance, especially in an aging population with frequent sleep disorders. Overall, it is fundamental to maintain and promote the correct functions of the muscle intrinsic clock machinery with the aim to protect against the loss of muscle during aging or in chronic disease conditions that could lead to muscle wasting. As a consequence, increased knowledge of the relationship between the skeletal muscle molecular clock and muscle-bone crosstalk may lead to a better understanding of aging-related diseases such as sarcopenia and osteoporosis. 

## References

[1] Bass J., Takahashi J.S. (2010). Circadian integration of metabolism and energetics. Science.

[2] Holzberg D., Albrecht U. (2003). The circadian clock: A manager of biochemical processes within the organism. J. Neuroendocrinol..

[3] Moore R.Y., Eichler V.B. (1972). Loss of a circadian adrenal corticosterone rhythm following suprachiasmatic lesions in the rat. Brain Res..

[4] Hoppeler H., Fluck M. (2002). Normal mammalian skeletal muscle and its phenotypic plasticity. J. Exp. Biol..

[5] Poole D.C. (1986). Measurements of the anaerobic work capacity in a group of highly trained runners. Med. Sci. Sports Exerc..

[6] Janssen I., Heymsfield S.B., Wang Z.M., Ross R. (2000). Skeletal muscle mass and distribution in 468 men and women aged 18–88 yr. J. Appl. Physiol. (1985).

[7] Deschenes M.R. (2004). Effects of aging on muscle fibre type and size. Sports Med..

[8] Silva A.M., Shen W., Heo M., Gallagher D., Wang Z., Sardinha L.B., Heymsfield S.B. (2010). Ethnicity-related skeletal muscle differences across the lifespan. Am. J. Hum. Biol..

[9] Fuggle N., Shaw S., Dennison E., Cooper C. (2017). Sarcopenia. Best Pract. Res. Clin. Rheumatol..

[10] Tournadre A., Vial G., Capel F., Soubrier M., Boirie Y. (2019). Sarcopenia. Joint Bone Spine.

[11] Cruz-Jentoft A.J., Landi F. (2014). Sarcopenia. Clin. Med..

[12] Ryall J.G., Schertzer J.D., Lynch G.S. (2008). Cellular and molecular mechanisms underlying age-related skeletal muscle wasting and weakness. Biogerontology.

[13] Doherty T.J. (2003). Invited review: Aging and sarcopenia. J. Appl. Physiol. (1985).

[14] Lexell J., Taylor C.C., Sjöström M. (1988). What is the cause of the ageing atrophy? Total number, size and proportion of different fiber types studied in whole vastus lateralis muscle from 15- to 83-year-old men. J. Neurol. Sci..

[15] Doherty T.J., Brown W.F. (1997). Age-related changes in the twitch contractile properties of human thenar motor units. J. Appl. Physiol. (1985).

[16] Tomlinson B.F., Irving D. (1977). The numbers of limb motor neurons in the human lumbosacral cord throughout life. J. Neurol. Sci..

[17] Leger B., Cartoni R., Praz M., Lamon S., Deriaz O., Crettenand A., Gobelet C., Rohmer P., Konzelmann M., Luthi F. (2006). Akt signalling through GSK-3beta, mTOR and Foxo1 is involved in human skeletal muscle hypertrophy and atrophy. J. Physiol..

[18] Argiles J.M., Busquets S., Stemmler B., Lopez-Soriano F.J. (2014). Cancer cachexia: Understanding the molecular basis. Nat. Rev. Cancer.

[19] Dhillon R.J., Hasni S. (2017). Pathogenesis and Management of Sarcopenia. Clin. Geriatr. Med..

[20] Newman A.B., Kupelian V., Visser M., Simonsick E.M., Goodpaster B.H., Kritchevsky S.B., Rubin S.M., Harris T.B. (2006). Strength, but not muscle mass, is associated with mortality in the health, aging and body composition study cohort. J. Gerontol. A Biol. Sci. Med. Sci..

[21] Studenski S.A., Peters K.W., Alley D.E., Cawthon P.M., McLean R.R., Harris T.B., Ferrucci L., Guralnik J.M., Fragala M.S., Kenny A.M. (2014). The FNIH sarcopenia project: Rationale, study description, conference recommendations, and final estimates. J. Gerontol. A Biol. Sci. Med. Sci..

[22] Cheung C.L., Lam K.S.L., Cheung B.M.Y. (2016). Evaluation of cutpoints for low lean mass and slow gait speed in predicting death in the National Health and Nutrition Examination Survey 1999–2004. J. Gerontol. A Biol. Sci. Med. Sci..

[23] Bischoff-Ferrari H.A., Orav J.E., Kanis J.A., Rizzoli R., Schlögl M., Staehelin H.B., Willett W.C., Dawson-Hughes B. (2015). Comparative performance of current definitions of sarcopenia against the prospective incidence of falls among community-dwelling seniors age 65 and older. Osteoporos. Int..

[24] Baumgartner R.N., Wayne S.J., Waters D.L., Janssen I., Gallagher D., Morley J.E. (2004). Sarcopenic obesity predicts instrumental activities of daily living disability in the elderly. Obes. Res..

[25] Beaudart C., Zaaria M., Pasleau F., Reginster J.Y., Bruyère O. (2017). Health outcomes of sarcopenia: A systematic review and meta-analysis. PLoS ONE.

[26] Sousa A.S., Guerra R.S., Fonseca I., Pichel F., Ferreira S., Amaral T.F. (2016). Financial impact of sarcopenia on hospitalization costs. Eur. J. Clin. Nutr..

[27] Von Haehling S., Morley J.E., Anker S.D. (2010). An overview of sarcopenia: Facts and numbers on prevalence and clinical impact. J. Cachexia Sarcopenia Muscle.

[28] Patel H.P., Syddall H.E., Jameson K., Robinson S., Denison H., Roberts H.C., Edwards M., Dennison E., Cooper C., Aihie Sayer A. (2013). Prevalence of sarcopenia in community-dwelling older people in the UK using the European Working Group on Sarcopenia in Older People (EWGSOP) definition: Findings from the Hertfordshire Cohort Study (HCS). Age Ageing.

[29] Brown J.C., Harhay M.O., Harhay M.N. (2016). Sarcopenia and mortality among a population-based sample of community-dwelling older adults. J. Cachexia Sarcopenia Muscle.

[30] Kim H., Hirano H., Edahiro A., Ohara Y., Watanabe Y., Kojima N., Kim M., Hosoi E., Yoshida Y., Yoshida H. (2016). Sarcopenia: Prevalence and associated factors based on different suggested definitions in community-dwelling older adults. Geriatr. Gerontol. Int..

[31] Wu I.C., Lin C.C., Hsiung C.A., Wang C.Y., Wu C.H., Chan D.C., Li T.C., Lin W.Y., Huang K.C., Chen C.Y. (2014). Sarcopenia and Translational Aging Research in Taiwan Team. Epidemiology of sarcopenia among community-dwelling older adults in Taiwan: A pooled analysis for a broader adoption of sarcopenia assessments. Geriatr. Gerontol. Int..

[32] Messina C., Maffi G., Vitale J.A., Ulivieri F.M., Guglielmi G., Sconfienza L.M. (2018). Diagnostic imaging of osteoporosis and sarcopenia: A narrative review. Quant. Imaging Med. Surg..

[33] Cesari M., Fielding R.A., Pahor M., Goodpaster B., Hellerstein M., Van Kan G.A., Anker S.D., Rutkove S., Vrijbloed J.W., Isaac M. (2012). International Working Group on Sarcopenia. Biomarkers of sarcopenia in clinical trials-recommendations from the International Working Group on Sarcopenia. J. Cachexia Sarcopenia Muscle.

[34] Baumgartner R.N., Koehler K.M., Gallagher D., Romero L., Heymsfield S.B., Ross R.R., Garry P.J., Lindeman R.D. (1998). Epidemiology of sarcopenia among the elderly in New Mexico. Am. J. Epidemiol..

[35] Muscaritoli M., Anker S.D., ArgileÂs J., Aversa Z., Bauer J.M., Biolo G., Boirie Y., Bosaeus I., Cederholm T., Costelli P. (2010). Consensus definition of sarcopenia, cachexia and pre-cachexia: Joint document elaborated by Special Interest Groups (SIG) “cachexia-anorexia in chronic wasting diseases” and “nutrition in geriatrics”. Clin. Nutr..

[36] Cruz-Jentoft A.J., Baeyens J.P., Bauer J.M., Boirie Y., Cederholm T., Landi F., Martin F.C., Michel J.P., Rolland Y., Schneider S.M. (2010). Sarcopenia: European consensus on definition and diagnosis: Report of the European Working Group on Sarcopenia in Older People. Age Ageing.

[37] Fielding R.A., Vellas B., Evans W.J., Bhasin S., Morley J.E., Newman A.B., Abellan van Kan G., Andrieu S., Bauer J., Breuille D. (2011). Sarcopenia: An undiagnosed condition in older adults. Current consensus definition: Prevalence, etiology, and consequences. International working group on sarcopenia. J. Am. Med. Dir. Assoc..

[38] Dam T.T., Peters K.W., Fragala M., Cawthon P.M., Harris T.B., McLean R., Shardell M., Alley D.E., Kenny A., Ferrucci L. (2014). An evidence-based comparison of operational criteria for the presence of sarcopenia. J. Gerontol. A Biol. Sci. Med. Sci..

[39] Cao L., Morley J.E. (2016). Sarcopenia Is Recognized as an Independent Condition by an International Classification of Disease, Tenth Revision, Clinical Modification (ICD-10-CM) Code. J. Am. Med. Dir. Assoc..

[40] Billiard M. (2003). Sleep Physiology, Investigations, and Medicine.

[41] Halberg F., Carandente F., Cornelissen G., Katinas G.S. (1977). Glossary of chronobiology (author’s transl). Chronobiologia.

[42] Koukkari W.L., Southern R.B. (2006). Introducing Biological Rhythms/a Primer on the Temporal Organization of Life, with Implications for Health, Societies, Reproduction and the Natural Environment.

[43] Deprins J., Cornelissen G., Halberg F. (1977). Harmonic interpolation on equispaced series. Chronobiologia.

[44] Reilly T., Waterhouse J. (2009). Sports performance: Is there evidence that the body clock plays a role?. Eur. J. Appl. Physiol..

[45] Yoo S.H., Yamazaki S., Lowrey P.L., Shimomura K., Ko C.H., Buhr E.D., Siepka S.M., Hong H.K., Oh W.J., Yoo O.J. (2004). PERIOD2: LUCIFERASE real-time reporting of circadian dynamics reveals persistent circadian oscillations in mouse peripheral tissues. Proc. Natl. Acad. Sci. USA.

[46] Takahashi J.S., Hong H.K., Ko C.H., McDearmon E.L. (2008). The genetics of mammalian circadian order and disorder: Implications for physiology and disease. Nat. Rev. Genet..

[47] Schibler U. (2009). The 2008 Pittendrigh/Aschoff lecture: Peripheral phase coordination in the mammalian circadian timing system. J. Biol. Rhythm..

[48] Damiola F., Le Minh N., Preitner N., Kornmann B., Fleury-Olela F., Schibler U. (2000). Restricted feeding uncouples circadian oscillators in peripheral tissues from the central pacemaker in the suprachiasmatic nucleus. Genes Dev..

[49] Ko C.H., Takahashi J.S. (2006). Molecular components of the mammalian circadian clock. Hum. Mol. Genet..

[50] Kalsbeek A., Palm I.F., La Fleur S.E., Scheer F.A., Perreau-Lenz S., Ruiter M., Kreier F., Cailotto C., Buijs R.M. (2006). SCN outputs and the hypothalamic balance of life. J. Biol. Rhythm..

[51] Schibler U., Naef F. (2005). Cellular oscillators: Rhythmic gene expression and metabolism. Curr. Opin. Cell Biol..

[52] Dibner C., Schibler U., Albrecht U. (2010). The mammalian circadian timing system: Organization and coordination of central and peripheral clocks. Annu. Rev. Physiol..

[53] Van Moorsel D., Hansen J., Havekes B., Scheer F.A.J.L., Jörgensen J.A., Hoeks J., Schrauwen-Hinderling V.B., Duez H., Lefebvre P., Schaper N.C. (2016). Demonstration of a day-night rhythm in human skeletal muscle oxidative capacity. Mol. Metab..

[54] Zhang R., Lahens N.F., Balance H.I., Hughes M.E., Hogenesch J.B. (2014). A circadian gene expression atlas in mammals: Implications for biology and medicine. Proc. Natl. Acad. Sci. USA.

[55] Yamazaki S., Takahashi J.S. (2005). Real-time luminescence reporting of circadian gene expression in mammals. Methods Enzymol..

[56] Miller B.H., McDearmon E.L., Panda S., Hayes K.R., Zhang J., Andrews J.L., Antoch M.P., Walker J.R., Esser K.A., Hogenesch J.B. (2007). Circadian and CLOCK-controlled regulation of the mouse transcriptome and cell proliferation. Proc. Natl. Acad. Sci. USA.

[57] Chatterjee S., Ma K. (2016). Circadian clock regulation of skeletal muscle growth and repair. F1000Res..

[58] Bunger M.K., Wilsbacher L.D., Moran S.M., Clendenin C., Radcliffe L.A., Hogenesch J.B., Simon M.C., Takahashi J.S., Bradfield C.A. (2000). Mop3 is an essential component of the master circadian pacemaker in mammals. Cell.

[59] Kondratov R.V., Kondratova A.A., Gorbacheva V.Y., Vykhovanets O.V., Antoch M.P. (2006). Early aging and age-related pathologies in mice deficient in *Bmal*1, the core component of the circadian clock. Genes Dev..

[60] Yang G., Chen L., Grant G.R., Paschos G., Song W.L., Musiek E.S., Lee V., McLoughlin S.C., Grosser T., Cotsarelis G. (2016). Timing of expression of the core clock gene *Bmal*1 influences its effects on aging and survival. Sci. Transl. Med..

[61] Dyar K.A., Ciciliot S., Wright L.E., Biensø R.S., Tagliazucchi G.M., Patel V.R., Forcato M., Paz M.I., Gudiksen A., Solagna F. (2013). Muscle insulin sensitivity and glucose metabolism are controlled by the intrinsic muscle clock. Mol. Metab..

[62] Schiaffino S., Blaauw B., Dyar K.A. (2016). The functional significance of the skeletal muscle clock: lessons from *Bmal*1 knockout models. Skelet Muscle.

[63] Andrews J.L., Zhang X., McCarthy J.J., McDearmon E.L., Hornberger T.A., Russell B., Campbell K.S., Arbogast S., Reid M.B., Walker J.R. (2010). CLOCK and *Bmal*1 regulate MyoD and are necessary for maintenance of skeletal muscle phenotype and function. Proc. Natl. Acad. Sci. USA.

[64] Riley L.A., Esser K.A. (2017). The Role of the Molecular Clock in Skeletal Muscle and What It Is Teaching Us About Muscle-Bone Crosstalk. Curr. Osteoporos Rep..

[65] Schiaffino S., Dyar K.A., Ciciliot S., Blaauw B., Sandri M. (2013). Mechanisms regulating skeletal muscle growth and atrophy. FEBS J..

[66] Chatterjee S., Yin H., Nam D., Li Y., Ma K. (2015). Brain and muscle Arnt-like 1 promotes skeletal muscle regeneration through satellite cell expansion. Exp. Cell Res..

[67] Pallafacchina G., Blaauw B., Schiaffino S. (2013). Role of satellite cells in muscle growth and maintenance of muscle mass. Nutr. Metab. Cardiovasc. Dis..

[68] Lipton J.O., Yuan E.D., Boyle L.M., Ebrahimi-Fakhari D., Kwiatkowski E., Nathan A., Güttler T., Davis F., Asara J.M., Sahin M. (2015). The Circadian Protein *Bmal*1 Regulates Translation in Response to S6K1-Mediated Phosphorylation. Cell.

[69] Latres E., Amini A.R., Amini A.A., Griffiths J., Martin F.J., Wei Y., Lin H.C., Yancopoulos G.D., Glass D.J. (2005). Insulin-like growth factor-1 (IGF-1) inversely regulates atrophy-induced genes via the phosphatidylinositol 3-kinase/Akt/ mammalian target of rapamycin (PI3K/Akt/mTOR) pathway. J. Biol. Chem..

[70] Burke L., Downes M., Carozzi A., Giguère V., Muscat G.E. (1996). Transcriptional repression by the orphan steroid receptor RVR/Rev-erb beta is dependent on the signature motif and helix 5 in the E region: Functional evidence for a biological role of RVR in myogenesis. Nucleic Acids Res..

[71] Raichur S., Fitzsimmons R.L., Myers S.A., Pearen M.A., Lau P., Eriksson N., Wang S.M., Muscat G.E. (2010). Identification and validation of the pathways and functions regulated by the orphan nuclear receptor, ROR alpha1, in skeletal muscle. Nucleic Acids Res..

[72] Haus E., Smolensky M. (2006). Biological clocks and shift work: Circadian dysregulation and potential long-term effects. Cancer Causes Control.

[73] Deschenes M.R., Sharma J.V., Brittingham K.T., Casa D.J., Armstrong L.E., Maresh C.M. (1998). Chronobiological effects on exercise performance and selected physiological responses. Eur. J. Appl. Physiol. Occup. Physiol..

[74] Gauthier A., Davenne D., Martin A., Van Hoecke J. (2001). Time of day effects on isometric and isokinetic torque developed during elbow flexion in humans. Eur. J. Appl. Physiol..

[75] Drust B., Waterhouse J., Atkinson G., Edwards B., Reilly T. (2005). Circadian rhythms in sports performance—An update. Chronobiol. Int..

[76] Sedliak M., Finni T., Cheng S., Kraemer W.J., Häkkinen K. (2007). Effect of time-of-day-specific strength training on serum hormone concentrations and isometric strength in men. Chronobiol. Int..

[77] Vitale J.A., Weydahl A. (2017). Chronotype, Physical Activity, and Sport Performance: A Systematic Review. Sports Med..

[78] Vitale J.A., Bonato M., La Torre A., Banfi G. (2019). Heart Rate Variability in Sport Performance: Do Time of Day and Chronotype Play A Role?. J. Clin. Med..

[79] Vitale J.A., Banfi G., La Torre A., Bonato M. (2018). Effect of a Habitual Late-Evening Physical Task on Sleep Quality in Neither-Type Soccer Players. Front. Physiol..

[80] Wolff G., Esser K.A. (2012). Scheduled exercise phase shifts the circadian clock in skeletal muscle. Med. Sci. Sports Exerc..

[81] Marchant E.G., Mistlberger R.E. (1996). Entrainment and phase shifting of circadian rhythms in mice by forced treadmill running. Physiol. Behav..

[82] Vitale J.A., Lombardi G., Weydahl A., Banfi G. (2018). Biological rhythms, chronodisruption and chrono-enhancement: The role of physical activity as synchronizer in correcting steroids circadian rhythm in metabolic dysfunctions and cancer. Chronobiol. Int..

[83] Angelousi A., Kassi E., Nasiri-Ansari N., Weickert M.O., Randeva H., Kaltsas G. (2018). Clock genes alterations and endocrine disorders. Eur. J. Clin. Investig..

[84] Saracino P.G., Rossetti M.L., Steiner J.L., Gordon B.S. (2019). Hormonal regulation of core clock gene expression in skeletal muscle following acute aerobic exercise. Biochem. Biophys. Res. Commun..

[85] Bambaeichi E., Reilly T., Cable N.T., Giacomoni M. (2005). Influence of time of day and partial sleep loss on muscle strength in eumenorrheic females. Ergonomics.

[86] Zambon A.C., McDearmon E.L., Salomonis N., Vranizan K.M., Johansen K.L., Adey D., Takahashi J.S., Schambelan M., Conklin B.R. (2003). Time- and exercise-dependent gene regulation in human skeletal muscle. Genome Biol..

[87] Nakao R., Yamamoto S., Horikawa K., Yasumoto Y., Nikawa T., Mukai C., Oishi K. (2015). Atypical expression of circadian clock genes in denervated mouse skeletal muscle. Chronobiol. Int..

[88] Dyar K.A., Ciciliot S., Tagliazucchi G.M., Pallafacchina G., Tothova J., Argentini C., Agatea L., Abraham R., Ahdesmäki M., Forcato M. (2015). The calcineurin-NFAT pathway controls activity-dependent circadian gene expression in slow skeletal muscle. Mol. Metab..

[89] Stokkan K.A., Yamazaki S., Tei H., Sakaki Y., Menaker M. (2001). Entrainment of the circadian clock in the liver by feeding. Science.

[90] Panda S., Antoch M.P., Miller B.H., Su A.I., Schook A.B., Straume M., Schultz P.G., Kay S.A., Takahashi J.S., Hogenesch J.B. (2002). Coordinated transcription of key pathways in the mouse by the circadian clock. Cell.

[91] Karatsoreos I.N., Bhagat S., Bloss E.B., Morrison J.H., McEwen B.S. (2011). Disruption of circadian clocks has ramifications for metabolism, brain, and behavior. Proc. Natl. Acad. Sci. USA.

[92] Pan A., Schernhammer E.S., Sun Q., Hu F.B. (2011). Rotating night shift work and risk of type 2 diabetes: Two prospective cohort studies in women. PLoS Med..

[93] Parkes K.R. (2002). Shift work and age as interactive predictors of body mass index among offshore workers. Scand. J. Work Environ. Health.

[94] Hodge B.A., Wen Y., Riley L.A., Zhang X., England J.H., Harfmann B.D., Schroder E.A., Esser K.A. (2015). The endogenous molecular clock orchestrates the temporal separation of substrate metabolism in skeletal muscle. Skelet. Muscle.

[95] Dyar K.A., Hubert M.J., Mir A.A., Ciciliot S., Lutter D., Greulich F., Quagliarini F., Kleinert M., Fischer K., Eichmann T.O. (2018). Transcriptional programming of lipid and amino acid metabolism by the skeletal muscle circadian clock. PLoS Biol..

[96] Wada T., Ichihashi Y., Suzuki E., Kosuge Y., Ishige K., Uchiyama T., Makishima M., Nakao R., Oishi K., Shimba S. (2018). Deletion of *Bmal*1 Prevents Diet-Induced Ectopic Fat Accumulation by Controlling Oxidative Capacity in the Skeletal Muscle. Int. J. Mol. Sci..

[97] Duez H., Staels B. (2008). The nuclear receptors Rev-erbs and RORs integrate circadian rhythms and metabolism. Diabetes Vasc. Dis. Res..

[98] Yin L., Wu N., Lazar M.A. (2010). Nuclear receptor Rev-erbalpha: A heme receptor that coordinates circadian rhythm and metabolism. Nucl. Recept. Signal..

[99] Delezie J., Dumont S., Dardente H., Oudart H., Gréchez-Cassiau A., Klosen P., Teboul M., Delaunay F., Pévet P., Challet E. (2012). The nuclear receptor REV-ERBα is required for the daily balance of carbohydrate and lipid metabolism. FASEB J..

[100] Woldt E., Sebti Y., Solt L.A., Duhem C., Lancel S., Eeckhoute J., Hesselink M.K., Paquet C., Delhaye S., Shin Y. (2013). Rev-erb-α modulates skeletal muscle oxidative capacity by regulating mitochondrial biogenesis and autophagy. Nat. Med..

[101] Azmi S., Ozog A., Taneja R. (2004). Sharp-1/DEC2 inhibits skeletal muscle differentiation through repression of myogenic transcription factors. J. Biol. Chem..

[102] Cho H., Zhao X., Hatori M., Yu R.T., Barish G.D., Lam M.T., Chong L.W., DiTacchio L., Atkins A.R., Glass C.K. (2012). Regulation of circadian behaviour and metabolism by REV-ERB-α and REV-ERB-β. Nature.

[103] McDearmon E.L., Patel K.N., Ko C.H., Walisser J.A., Schook A.C., Chong J.L., Wilsbacher L.D., Song E.J., Hong H.K., Bradfield C.A. (2006). Dissecting the functions of the mammalian clock protein *Bmal*1 by tissue-specific rescue in mice. Science.

